# Persistent left superior vena cava in atrial septal defect sinus venosus type: diagnosis with saline contrast echocardiography—a case series

**DOI:** 10.1002/ccr3.883

**Published:** 2017-03-17

**Authors:** Purwati Pole Rio, Hasanah Mumpuni, Dyah Wulan Anggrahini, Lucia Kris Dinarti

**Affiliations:** ^1^ Department of Cardiology and Vascular MedicineFaculty of MedicineUniversitas Gadjah Mada,‐Sardjito General HospitalYogyakartaIndonesia

**Keywords:** Atrial septal defect, persistent left superior vena cava, saline contrast echocardiography, transthoracic echocardiography

## Abstract

Persistent Left Superior Vena Cava (PLSVC) should be suspected if we find dilatation of coronary sinus. Sophisticated imaging is not always available in each health care provider. Transthoracic echocardiography (TTE) with agitated saline injections through the left and right antecubital veins provides a simple, and inexpensive, but effective study for a rapid bedside diagnosis of PSLVC.

Saline contrast echocardiography is an established imaging modality, where logical interpretation of a carefully performed examination is very important. Saline contrast echocardiography has commonly been used to diagnose patent foramen ovale 1. Unfortunately, this imaging tool is still under utilized to diagnose other intra‐ and extracardiac shunts. This case series will highlight one of the important roles of saline contrast echocardiography, which is diagnosing the Persistent Left Superior Vena Cava (PLSVC), a very rare congenital condition that has important clinical implications.

## Case 1

**Figure 1:**
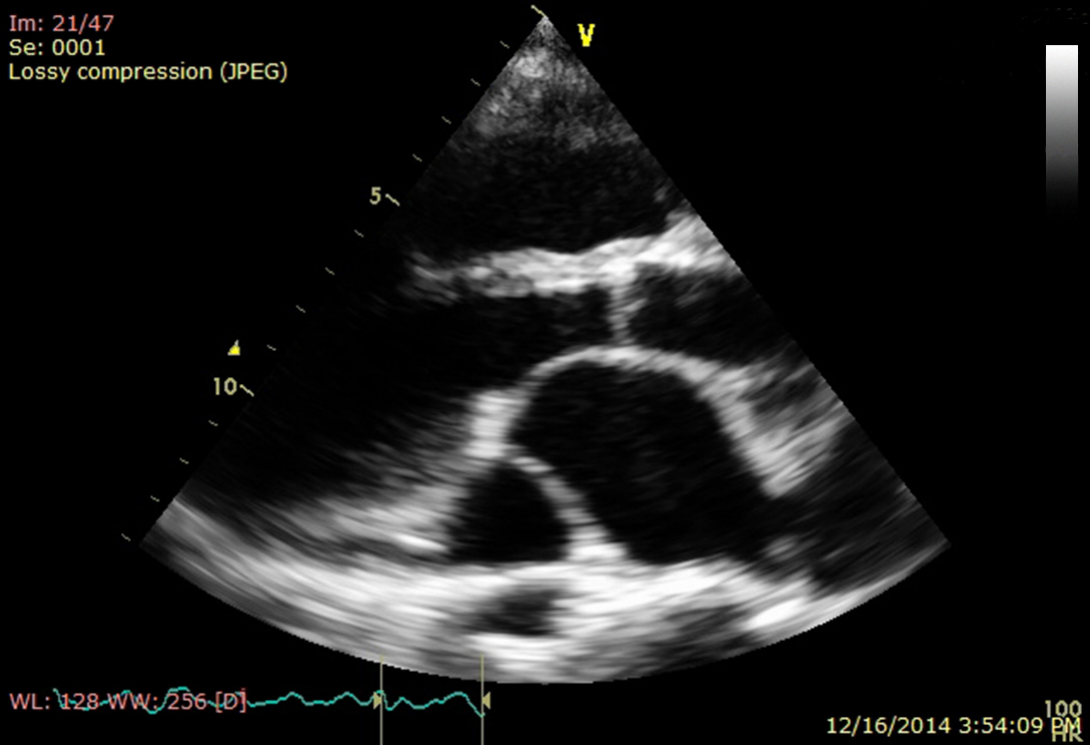
Transthoracic echocardiography, parasternal long axis view, showing dilatation of coronary sinus.

**Figure 2:**
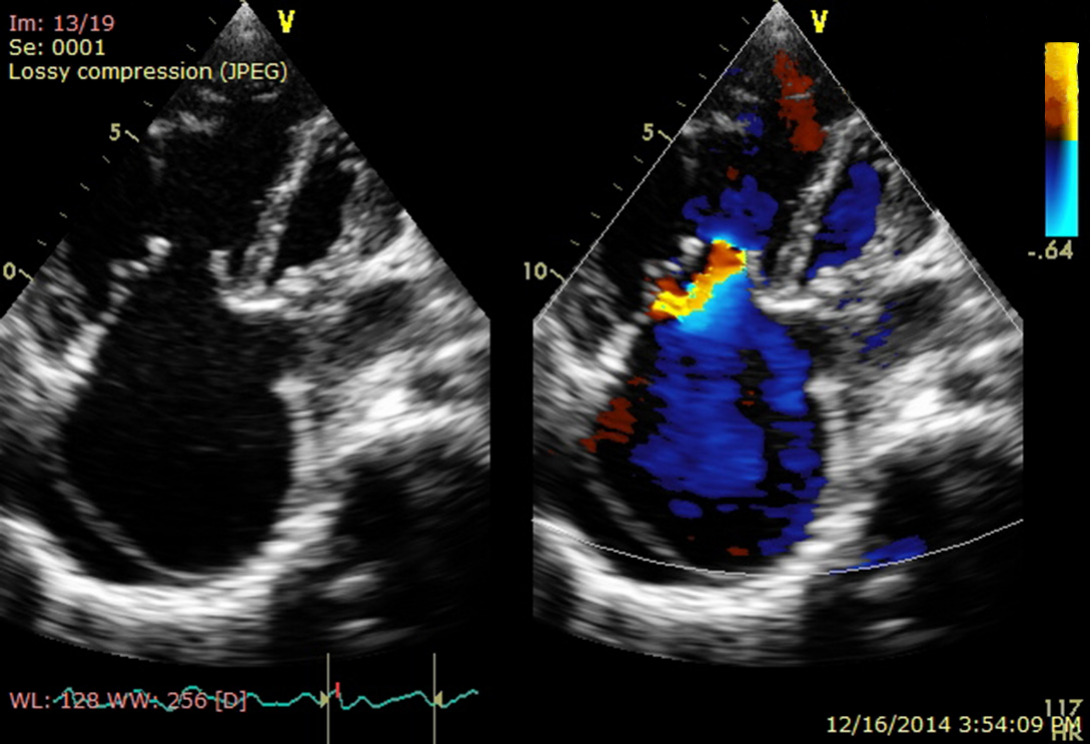
Transthoracic echocardiography, apical four‐chamber view, showing atrial septal defect sinus venosus type, dilatation of right atrium, right ventricle, and coronary sinus.

A female, 51 years old, came with chief complaints of being easily fatigued, shortness of breath, and sometimes suffering from chest discomfort. These symptoms started happening 6 months ago. In the thoracal examination, we found cardiomegaly (enlarged heart) with right ventricular heaving, constant S2 split, hardening P2, and pansystolic murmur three of six in left parasternal line of intercostals space III–IV. Electrocardiography study revealed right axis deviation with right ventricular enlargement. Chest X‐ray revealed enlargement of right atria and right ventricle. The TTE revealed ASD of the sinus venosus type ranging 30–33 mm, and dilatation of coronary sinus with diameter of 27 mm (Figs 1 and 2).

**Figure 3:**
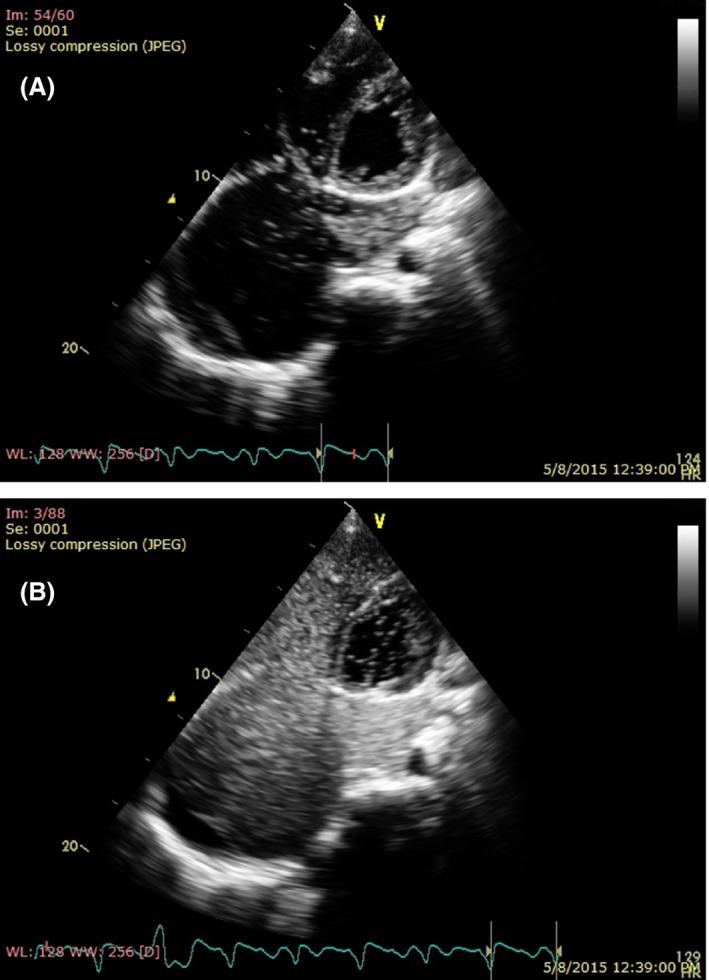
(A, B) Injection of saline bubble from left cubital vein showing bubble appear in the sinus coronarius and then right atrium.

Dilatation of the coronary sinus raised suspicion of PLSVC. The TTE revealed echo drop out in the interatrial septum with diameter of 4 cm, followed with dilatation of the coronary sinus (34 mm). A saline contrast echo was then performed. Imagery after injection of saline bubble from left cubital vein showed bubbles entering the coronary sinus and then the right atrium. (Fig. 3).

**Figure 4:**
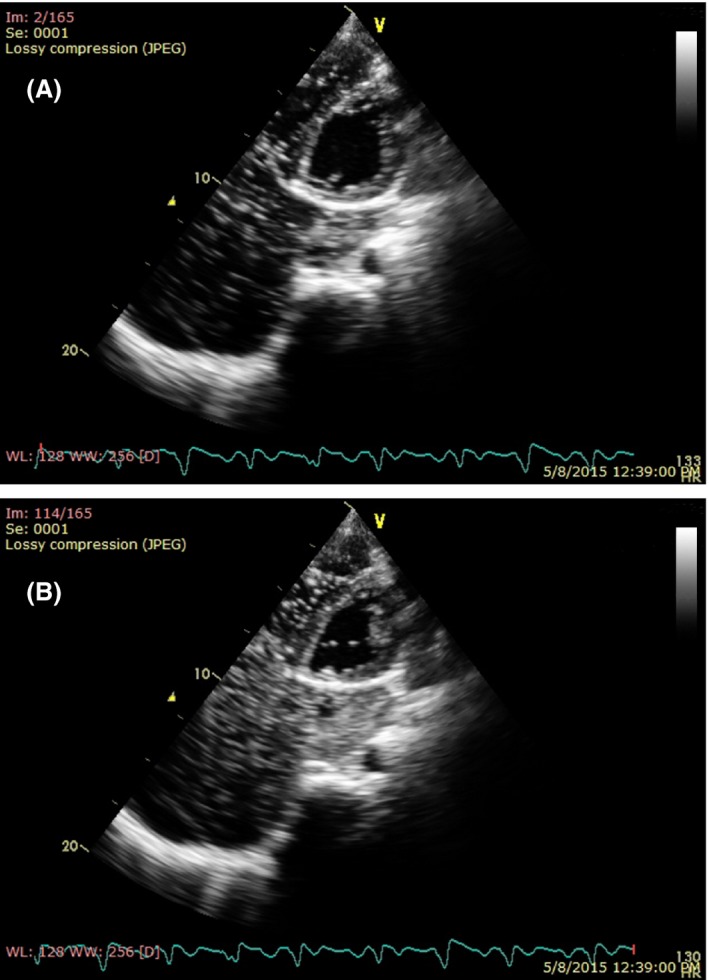
(A, B) Injection of bubble saline from right cubital vein showing bubble traveling appear in the right atrium before the sinus coronarius.

When injection of saline bubble was performed from right cubital vein, the echo contrast enhanced the right atrium before the coronary sinus, suggesting PLSVC with normal right SVC (Fig. 4).

**Figure 5:**
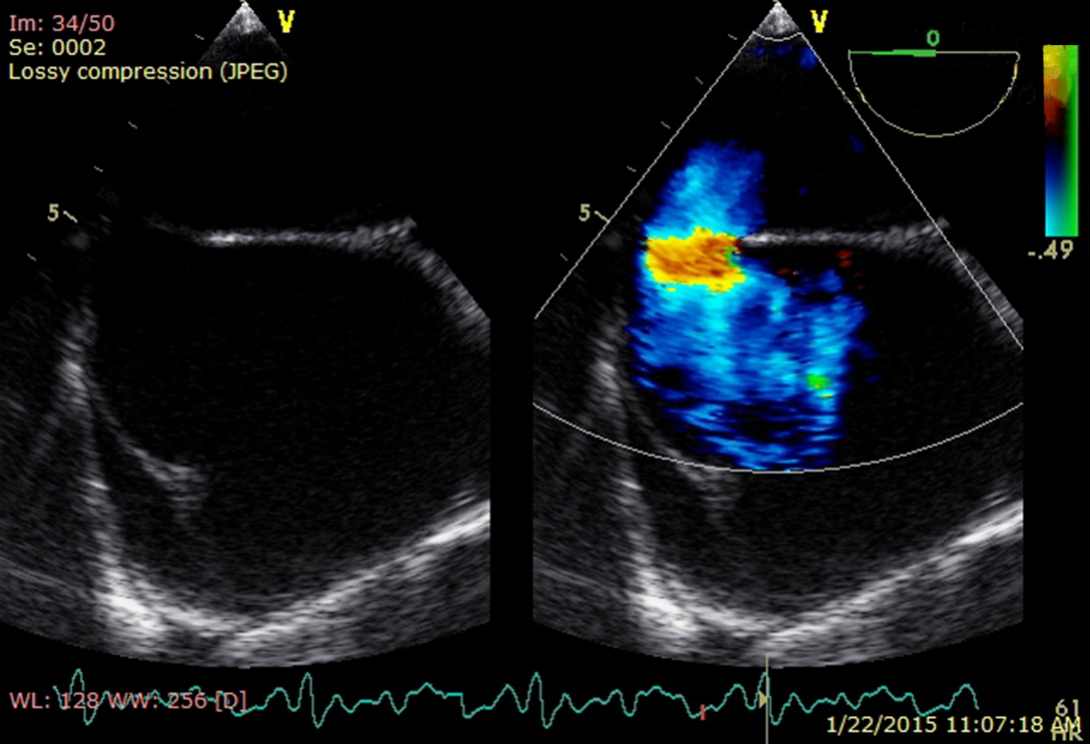
Transesophageal echocardiography showing Atrial Septal Defect sinus venosus type.

Transesophageal echocardiography was done showing atrial septal defect of the sinus venosus type (Fig. 5).

## Case 2

A male, 31 years old, came with chief complaint of chest pain after strenuous activity that started 10 months ago. From the examination, we found cardiomegaly (enlarged heart), right ventricular heaving, and S2 constant split with systolic murmur two of six in the left parasternal line of intercostal space III–IV. Chest X‐rays showed right ventricular enlargement with bulging of the pulmonary segment. TTE shows ASD of the sinus venosus type with a left to right shunt with diameter of 22–25 mm, and dilatation of coronary sinus with diameter of 26 mm. Dilatation of the coronary sinus raised suspicion of PLSVC. This examination was then continued with saline contrast echocardiography. Imagery after injection of contrast from left cubital vein showed the bubbles traveling from the coronary sinus and then into right atrium shortly after the contrast was injected.

Imagery after injection of contrast from the right cubital vein showed bubbles entering the right atrium and then the coronary sinus. Right heart catheterization and MSCT confirmed the presence of PLSVC with small innominate veins.

## Discussion

Saline contrast echocardiography using agitated saline is an established, simple, inexpensive and yet surprisingly effective diagnostic tool that might help in solving a diagnostic problem 1. Although this examination has been used since 1968, the test is still under utilized in clinical practice 2. This examination provides a mapping of blood flow with the help of microbubbles, which is still useful even in the era of color flow Doppler studies. Saline contrast echocardiography can give more clear information, especially in a patient with a poor echo window or with other technical facts that can hamper the color flow imaging. While this approach may reduce the need for more expensive examinations such as MRI or cardiac CT, utilization of this test for diagnosing the PLSVC is still not commonly reported.

Persistent Left Superior Vena Cava is a rare thoraxic vein anomaly, occurring in 0.3–0.5% of the population 3. This anomaly occurs in 10% of patients with congenital heart disease 4. PLSVC occurs when the Marshall ligament fails to regress, resulting in left vein drain into the right atrium 5. In 92% of the cases, PLSVC drains to the right atrium via the coronary sinus. In 8% of the cases, PLSVC drains directly to the left atrium via the pulmonary vein, causing the right to left shunt 6. In this case series, both patients have PLSVC that drains into right atrium via the coronary sinus. In both cases, TTE reveals ASD with dilatation of the coronary sinus. In dilatation of the coronary sinus, we should always consider the possibility of PLSVC or defect of the coronary sinus; therefore, a saline contrast echocardiography must always be performed 7. Injection of bubble saline from the right cubital vein shows contrast traveling from the superior vena cava to the right atrium into the sinus coronarius suggesting the presence of a normal right superior vena cava. Injection of contrast from the left cubital vein shows bubbles traveling from the sinus coronarius into the right atrium suggesting PLSVC. There are several side effects of the saline test such as nausea, cough, dyspnoea, and transient paresthesia or parese that very rarely occur 8. Diagnosing PLSVC has important clinical implications such as in permanent pacemaker implantation or in canulation in open thorax surgery for correction of a congenital heart defect and Coronary Artery Bypass Graft or CABG 9, 10.

## Conclusion

Persistent Left Superior Vena Cava should be suspected if imagery reveals dilatation of the coronary sinus. Diagnosis can be easily made by adding saline contrast, where injection of contrast from the left cubital vein shows bubbles appearing in the coronary sinus before the right atrium. TTE with agitated saline injections through the left and right antecubital veins provides a simple, and inexpensive, but effective study for a rapid bedside diagnosis of PSLVC.

## Authorship

PPR: Contributed to writing the content and editing the figure attached. HM: Contributed to the idea, performing the transthoracic, and transesophageal echocardiography. DWA: Contributed by doing the final editing and translation. LKD: Contributed to the idea, editing content, the doctor in charge for the patients.

## Conflict of Interest

None declared.
